# Supplementary material for: Patterns of Intron Gain and Loss in Fungi
Source: PLoS Biol. 2004 Nov 30;2(12):e422. doi: 10.1371/journal.pbio.0020422 (PMC532390; doi:10.1371/journal.pbio.0020422)
Supplement: Table S1 — Also available at http://genes.mit.edu/NielsenEtAl/. (4.3 MB ZIP). [file pbio.0020422.st001.zip › NielsenEtAl/html/228.html]

AN1137.1.NCU06025.1.MG07781.1.FG05704.1


```
 CLUSTAL W (1.82) Multiple Sequence Alignments - Introns Inserted


Sequence 1: NCU06025.1	321 aa
Sequence 2: MG07781.1	326 aa
Sequence 3: FG05704.1	323 aa
Sequence 4: AN1137.1	329 aa
Alignment Length: 357 aa
Number Identitical Residues: 107 aa
Alignment Score (without introns) 6230


MG07781.1 	MASATATT-----------TEQPLLDADLAASVASVDRHGYLFGQKITHSLSPLFHSTIY
NCU06025.1	MSTATTTTSATTTMSVVQPRQQELLHLTSTPDITPYTRHGYLFGQKLAASMSPLLHSIVY
FG05704.1 	MSVQQITS-------------SPTGDSLQTSQVSQLERHGYLFGKKLTASLSPFLHDVIY
AN1137.1  	MEPITIPT--------------------------DRDGVAYLYGHPLRNSLSPPLHQTVY
          	*     .:                               .**:*: :  *:** :*. :*

MG07781.1 	QELGLRWEQFRLD---------SSDVSRFLQLMR-DPRFYG~ASVTMPNKVAIMPHLDHL
NCU06025.1	SHLSLNWAQLRLD---------SPSIPLFLQLAQ-HPDFYG~ASVTMPHKVAIIPHLDHL
FG05704.1 	KALGYNWGQVRLD---------SADIDGFLQLAQ-HPDFYG1ASVTMPNKVAIIPYLDEL
AN1137.1  	NALGLNWTQIPLSTATGTSFTRSPEISTFLSSVRSNPKFVG~SSVTMPWKVAIMPHLDDL
          	. *. .* *. *.:::.:: : *..:  **.  ::.* * * :***** ****:*:**.*

MG07781.1 	TDECRDVGACNTVFLRPDPSVPGGRIYCGANTDVIGVRESFLRNVADPDAVIRNRPALVL
NCU06025.1	TPECRDVGACNTLFLKTDPAT-GRRLYCGANTDVIGVRESFVQNVSDPARVYESRPALVI
FG05704.1 	TEECRDVGACNTLYLR---ERDGRRIFCGANTDVIGIRDSFYQNIESPDVVFHNKPALVV
AN1137.1  	TEDARQAGACNTIYLRK--EDDGKTQYVGTNTDCIGIREALLQGSPNGAEHFKGKPALIV
          	* :.*:.*****::*:      *   : *:*** **:*::: :.  .     ..:***::

MG07781.1 	GGGGAARSAVYALRRWLGVRDIYMVNRDAREVADVVADCTARGYG--QGLVHVASVEQAA
NCU06025.1	GGGGAARSAVYALHKWLGATDIYLVNRDKSEVDAVIAECTERGYG--DRLVHVASVEQAE
FG05704.1 	GGGGAARSAIYALRKKMQATTIYLVNRDEAEVEAVIADCVSRGYG--EGLLHVKTVAQAS
AN1137.1  	GGGGTARTAIYVLRKWLGVSKIYIVNRDAKEVEAILAEDKQRNPSPQVALVPVSDPSAAA
          	****:**:*:*.*:: : .  **:****  **  ::*:   *. ...  *: *     * 

MG07781.1 	ALEAPGAMVLCVPDFAPKTAEEVVARDVMSVFFAKE----RKGAILEMCYNPTPYTQLWA
NCU06025.1	GLEGPGAIVACIPDFPPKTEKEMLVRRIVETFLMKE----EKGAMLEMCYNPSPFTELGA
FG05704.1 	SLEAPGAIVACVPDFEPVTEEEIMARAVTEAFLDKP----QKGAILEMCYNPTPFTKLGA
AN1137.1  	TLEAPVAVVSGIPNYPPQTEEEIRARETLRVFLNRQTHEKDQGVILEMCYHPLPWTDIAQ
          	 **.* *:*  :*:: * * :*: .*    .*: : : .. :*.:*****:* *:*.:  

MG07781.1 	LAEGQGWQVILGTEALIYQGLEQ0DKYWTGKEVDDLP--VQKVTEVIASKLGQKPKL--
NCU06025.1	LAEHEGWQVILGTEALIWQGIEQ~VCIRP------------ELTLYCGGQLN-------
FG05704.1 	IAEDKGWKVILGTEALIYQGLEQ~DKYWTGKTLEEMP--IEKVHIAIAEKVAQRAEAKL
AN1137.1  	IAQDARWKVILGSEALIWQGLEQ~ARLWTGKDVVSEPGLVEKVQAFVAQTIAERSKSNL
          	:*:   *:****:****:**:**     ...   . ..  .::    .  : . .. .
```
